# Can the Effects of Exercise Therapy on Achilles Tendinopathy Be Enhanced by Adding Nutritional Advice—A Randomized Controlled Pilot Study

**DOI:** 10.3390/nu18101519

**Published:** 2026-05-10

**Authors:** Fanji Qiu, Bernd Wolfarth, Kirsten Legerlotz

**Affiliations:** 1 School of Sports Science and Physical Education, Nanjing Normal University, Wenyuan Road 1, Nanjing 210023, China; 2 Movement Biomechanics, Institute of Sport Sciences, Humboldt-Universität zu Berlin, Unter den Linden 6, 10099 Berlin, Germany; 3 Institute of Sport Sciences, Humboldt-Universität zu Berlin, Unter den Linden 6, 10099 Berlin, Germany; bernd.wolfarth@charite.de; 4 Department of Sports Medicine, Charité-Universitätsmedizin Berlin, 10117 Berlin, Germany; 5 Department of Movement and Training Sciences, Institute of Sport Sciences, University of Wuppertal, Gaußstraße 20, 42119 Wuppertal, Germany; legerlotz@uni-wuppertal.de

**Keywords:** nutrition advice, Achilles tendinopathy, exercise, RCT, resistance training

## Abstract

**Background:** The progression of orthopedic diseases such as rheumatism and tendinopathies can be affected by metabolic conditions. Recent research suggests that changes in nutrition may affect symptom severity and recovery in orthopedic diseases. This study aims to explore whether the therapeutic efficacy of exercise therapy can be enhanced by adding nutritional advice in Achilles tendinopathy. **Method:** This 12-week randomized controlled pilot trial enrolled 16 adult patients (age 39.38 ± 9.46 years) suffering from chronic Achilles tendinopathy (≥3 months of symptoms, Victorian Institute of Sport Assessment—Achilles (VISA-A) scores below 80). Participants were randomly assigned to either the experimental group, receiving nutritional advice combined with home-based high-load tendon exercise training, or the control group, receiving exercise training alone. Outcomes included VISA-A scores, visual analog scale (VAS) pain assessments, body composition, and blood markers, analyzed through both intention-to-treat and per-protocol approaches. **Results:** Baseline characteristics showed no significant intergroup differences. From pre to post VISA-A scores increased from 58.06 ± 12.06 to 74.51 ± 17.81 points (*p* = 0.005) and VAS decreased from 3.19 ± 2.32 to 1.55 ± 1.66 points (*p* = 0.048) across all participants. Within-group analysis demonstrated a significant VISA-A improvement (63.13 ± 10.08 to 81.39 ± 13.13 points) (*p* = 0.013) in the experimental group only. The control group experienced a significant increase of 6.74 ± 12.26 mmHg in diastolic blood pressure (*p* = 0.046). **Conclusions:** The exercise intervention improved functional and pain outcomes in all participants, with better VISA-A outcomes in the experimental group. However, a clearly superior effect of the combined strategy compared with exercise alone could not be detected in this pilot study with a limited sample size.

## 1. Introduction

Emerging research demonstrates that nutritional status significantly impacts musculoskeletal health. On the one hand, underfeeding and disordered eating patterns emerge as key contributors to lower extremity soft tissue injuries among female long-distance runners [1]. Similarly, nutritional deficiencies as a result of an inappropriate diet with low fat, vitamin, and mineral intake have been observed in female osteoporosis patients and are believed to worsen the condition [2]. On the other hand, overfeeding with a high-caloric and inappropriate diet may result in metabolic disorders [3,4,5], which are similarly known to adversely impact musculoskeletal health. This has been observed in diabetic patients aged ≥50 years with osteoporotic fractures, who exhibited a 2.8-fold higher risk of subsequent fractures compared to their non-diabetic counterparts [6]. Furthermore, population studies in middle-aged and older adults reveal significant positive correlations between insulin resistance markers (particularly the triglyceride-glucose index) and bone mineral density at the femoral neck, lumbar spine, and total hip regions, while showing inverse associations with osteopenia/osteoporosis prevalence [7]. Additionally, cohort data identify a positive relationship between the triglyceride to high-density lipoprotein ratio and incident gout risk in adult populations [8]. Therefore, it is conceivable that diet and metabolic disorders may also impact tendon health.

Specifically, metabolic diseases such as hypercholesterolemia and diabetes have been shown to impact tendon health and predispose individuals to tendinopathies, a common disorder characterized by chronic pain, swelling, and loss of function [9], which is associated with hypoechoic thickening and disorganized collagen fibers on ultrasound examination [10]. A cross-sectional study involving 5856 patients with an average age of 62 found that pre-diabetic individuals had a threefold increased risk of lower limb tendon injuries compared to those with normal HbA1c levels. Hypercholesterolemia was associated with a 1.5-fold increased risk of upper limb tendon injuries, and patients with metabolic syndrome had a 2.5-fold higher risk of tendon injuries [11]. The increased injury risk may arise from structural tendinous changes that are associated with metabolic diseases, as individuals with hypercholesterolemia had lower Achilles tendon stiffness and greater Achilles hysteresis, which was observed in a study comparing participants with hypercholesterolemia and diagnosed tendon xanthomas to healthy controls [12]. In addition to metabolic diseases, inflammatory processes may negatively affect Achilles tendon health, with chronic low-grade inflammation potentially leading to poor healing after acute tendon injuries [13] and elevated Interleukin-6 levels indicative of inflammatory processes being observed in Achilles tendinopathy patients [14,15]. Therefore, addressing metabolic diseases and reducing inflammatory processes by adjusting diet [16] may potentially aid in the treatment of Achilles tendinopathy.

Various treatment methods have been employed to manage Achilles tendinopathies, with exercise therapy being a crucial component. Eccentric training of the Achilles tendon has been applied since 1998 and has repeatedly been shown to foster functional recovery with significant pain reduction and restored activity levels [17,18,19]. Heavy Slow Resistance (HSR) training has also been used to treat Achilles tendinopathy, yielding results similar to those of eccentric training. Patients with chronic mid-portion Achilles tendinopathy showed significant improvements in VISA-A scores, pain reduction, and a decrease in tendon thickness and neovascularization after applying HSR training in a 12-week randomized controlled trial [18]. More recently, isometric high tendon loading has also proven effective in improving function in Achilles tendinopathy patients. After 12 weeks of home-based training, applying high tendon strain with high-load exercises at 90% of the maximum voluntary contraction, patients demonstrated significant increases in maximum plantar flexion strength, tendon stiffness, and VISA-A scores, along with notable pain reduction [20]. Hence, different exercise training methods effectively treat Achilles tendinopathy.

Furthermore, nutritional interventions can be applied in tendinopathy management, with the combination of nutritional supplementation and training being well established. Nutrients such as vitamin C may aid in the hydroxylation process and promote tendon healing, while vitamin D increases type I collagen mRNA levels and inhibits osteoblast differentiation and calcification in ligament fibroblasts. Diets rich in leucine and glycine may positively impact collagen synthesis in tendons [21]. Combining nutritional supplementation with exercise rehabilitation has proven effective in treating tendon diseases. Adolescent swimmers with overuse tendinopathy benefited from four weeks of creatine supplementation combined with isometric exercises, with pain reduction being more pronounced in the combined intervention group compared to the placebo group [22]. In patients with mid-portion Achilles tendinopathy, a combination of collagen peptide supplementation and calf-strengthening training over three months resulted in significant increases in VISA-A scores and a reduction in intratendinous microvessels [23]. While some ingredients of supplements, such as collagen, may be fully digested in the small intestine and thus not reach the tendon, others, such as possibly hydrolyzed collagen, may enter the circulation and accumulate in muscle or connective tissues, where they may stimulate collagen synthesis and matrix remodeling [24,25,26]. The underlying mechanism may involve the upregulation of key anabolic signaling pathways (PI3K-Akt), as observed in skeletal muscle by collagen peptide ingestion [27].

While there is evidence for the therapeutic effectiveness of nutritional supplementation in tendon disease, the effect of dietary counseling as a tool to improve treatment outcomes has not yet been investigated. However, even without directly controlling food intake, individual nutritional advice and its combination with exercise training can promote health. Improvements in diastolic blood pressure, hypersensitive C-reactive protein (hs-CRP), and workload were observed in obese adolescent girls after a three-month intervention program combining high-intensity interval training with nutritional advice [28]. It therefore remains unclear whether adding nutritional advice to exercise therapy can provide additional benefits in the treatment of Achilles tendinopathy, a research gap that our exploratory pilot trial is designed to address.

Thus, we aimed to investigate the therapeutic effectiveness of a treatment approach that combines exercise therapy with nutritional advice, hypothesizing that combining exercise therapy with nutritional advice will be more effective than exercise therapy alone in treating Achilles tendinopathy.

## 2. Materials and Methods

### 2.1. Study Design and Participants

This study was an exploratory, randomized controlled pilot trial, not powered as a confirmatory efficacy study. Subjects were recruited through social media and posters. They were first examined by orthopedic doctors from the sports medicine department and then, after a diagnosis of Achilles tendinopathy, provided with a validated, home-based training device for therapeutic strengthening exercises [29], instructed on how to perform the exercises [20], and randomly assigned to the control (exercise only) or experimental group (exercise and nutritional advice).

Included patients were 18–60-year-old adults with chronic Achilles tendinopathy (diagnosis by a doctor in the sports medicine outpatient clinic, verified by ultrasound) and a VISA-A score of less than 80 points, who had suffered from tendon pain for at least 3 months. During the anamnesis conducted by the medical doctor, the potential participants were asked about their medical history. Exclusion criteria included corticosteroid injections within 12 months prior to the study, use of antibiotics (especially fluoroquinolones), and lower extremity surgery. For patients reporting symptoms in both Achilles tendons, the leg with the lower VISA-A score and higher pain level was selected for the therapeutic intervention.

Twenty-six participants were interested in our study, of whom 10 were excluded because they did not meet the inclusion criteria. A total of 16 participants met the eligibility requirements and were finally enrolled in the study from March 2022 to March 2025. The subjects were randomly assigned to a control group (CG) or an experimental group (EG).

**Figure 1:**
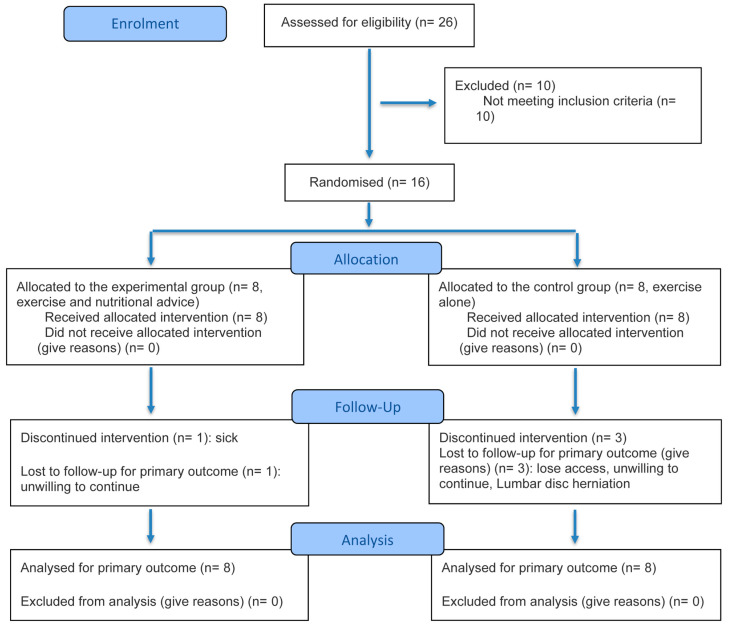
CONSORT flow diagram of participant recruitment and study design.

Ethics approval for this study was obtained from the Ethics Committee of the Charité—University Hospital Berlin (EA4/107/22). Informed written consent was obtained from all participants before starting the trial. The study was performed in compliance with the Declaration of Helsinki and the CONSORT (Consolidated Standards of Reporting Trials) guidelines [30] (Figure 1 and Supplemental File).

### 2.2. Randomization and Blinding

A set of random numbers was generated in Excel by the assistant researcher (J.N.), who safeguarded the allocation sequence. The numbers were placed in sequentially numbered, opaque, sealed envelopes and kept separately. Eligible participants were assigned an envelope containing a random number prior to enrollment. Participants with odd numbers were assigned to the experimental group, while those with even numbers were assigned to the control group.

Neither the medical doctors nor the patients involved in the recruitment and baseline assessment process were aware of the allocation sequence throughout the study. After completing baseline assessments and data collection at the clinic, the envelopes were opened, and the researcher (F.Q.) was informed of the group assignments. The timing of nutritional counseling differed between groups, with the experimental group receiving their nutritional advice soon after the baseline visit, whereas the control group was told that they would receive nutritional counseling only after completing all post-intervention assessments at week 12. All procedures of the study—group allocation, baseline measurements, interventions, and post-intervention measurements—strictly adhered to standardized assessment procedures. The study hypothesis and group allocations were not disclosed to any participants.

### 2.3. Interventions

All subjects performed the high-load tendon strengthening exercises over a period of 12 weeks, as described previously [20]. The exercise intervention utilized a validated home-based device that has previously been shown to stimulate the tendon (increasing cross-sectional area and tendon stiffness), strengthen the calf muscles, and reduce pain [20,29]. The training regimen consisted of four weekly sessions, each comprising five sets of four repetitions, with a one-minute rest between sets. Each repetition, specifically, each isometric plantar flexion contraction at 90% MVC, was held for 3 s, followed by 3 s of rest.

The treatment was the same for both groups, and the experimental group received nutritional advice from a sports medicine clinic nutritionist at the start of the 12-week intervention period, while the control group received nutritional advice after the intervention period. Since all participants received both exercise intervention and nutritional advice, they could not predict their group assignment. Before training, the patients received guidance on equipment use and the subjects’ maximum voluntary contraction (MVC) values were measured to determine the initial training load (90% of the average MVC value from five isometric plantar flexion contractions) [20]. The training programs were documented in a training diary [20]. The patients were allowed to maintain their usual exercise habits but were prohibited from additional calf plantar flexion strength training or introducing new types of lower limb training [20]. All patients were also offered 12 sessions of physical therapy focusing on passive treatments, avoiding any plantar flexion exercises as described previously [20].

### 2.4. Nutritional Advice

The patients recorded their diet for 7 days in a nutritional diary provided by the German Nutrition Society (DGE) [31]. A trained nutritionist assessed the participants’ recent dietary habits by analyzing the diaries. In a standardized in-person session lasting approximately 30 min, the nutritionist discussed the current diet with the patients and provided individualized dietary advice and recommendations for a healthy diet based on DGE guidelines, aimed at reducing systemic inflammation levels. This included e.g., advice to increase the amount of fruits and vegetables consumed and a reduction in processed food and sugar. After approximately 6 weeks, the patients received a follow-up phone call from the nutritionist for further counseling.

### 2.5. Adherence and Compliance

Exercise adherence was monitored using training diaries (blank templates are available in the Supplementary Files). Among participants who completed the 12-week intervention and provided post-intervention data, all completed the full Achilles tendon strengthening exercises as prescribed, corresponding to 100% adherence in the per-protocol population. In the ITT analysis, 5 participants were lost to follow-up and their missing outcomes were handled by multiple imputation. The uptake of any passive physiotherapy was not systematically recorded; however, this is unlikely to have influenced our outcomes, as a previous study reported no differences in clinical outcomes irrespective of the applied physiotherapeutic approach [14]. Compliance with the nutritional advice was reinforced by a follow-up telephone call from the dietitian at Week 6 of the nutritional intervention.

### 2.6. Outcomes

All outcomes were collected at baseline before the intervention as well as post-intervention. Primary outcomes were tendon function, which was assessed using VISA-A, a validated measure of Achilles tendinopathy severity [32] and pain, which was determined by asking the subjects their average value on a VAS (0–10 points) in the morning over the last seven days.

Secondary outcomes included variables derived from venous blood samples such as serum Interleukin-6 (IL-6), tumor necrosis factor alpha (TNF-α), C-reactive protein (CRP), glycated hemoglobin/hemoglobin A1c (HbA1c), total cholesterol, triglyceride (TRG), high-density lipoprotein (HDL), and low-density lipoprotein (LDL). The Vacuette heparin blood collection tubes were used for blood collection, including one EDTA (2 mL), fluoride plasma (2 mL) and one serum (8 mL). All samples were transported to a commercial provider of hospital laboratory diagnostics (Labor Berlin—Charité Vivantes GmbH, Berlin, Germany) for analysis according to standard procedures [14].

Anthropometric variables included age, sex, height, blood pressure (systolic blood pressure, SBP; diastolic blood pressure, DBP), body mass, fat-free mass, fat mass. Physical activity level was measured using the International Physical Activity Questionnaire (IPAQ) and reported as Met-min/week [33]. Body composition was measured via InBody (InBody Co., Ltd., Version 720, Berlin, Germany), as described previously [34].

Given the exploratory pilot nature of this trial with a limited sample size (*n* = 16), all analyses were considered exploratory and no formal a priori power calculation was performed. The primary outcomes (VISA-A and VAS) served as the main focus of inference, while all secondary outcomes and biomarker analyses were treated as hypothesis-generating. Therefore, secondary outcome results should be interpreted with caution due to the increased risk of false-positive findings.

### 2.7. Statistical Analysis

All statistical analyses were performed using SPSS 26 (SPSS Inc., Chicago, IL, USA). Statistical significance was set at a *p*-value less than 0.05. Descriptive statistics are presented as means ± SD for normally distributed continuous variables, and categorical variables are summarized as percentages.

The primary efficacy inference relied on between-group comparisons of post-intervention values, with VISA-A and VAS as the main outcomes. To assess baseline differences and evaluate the effects of two treatment methods on VISA-A scores, pain levels, and blood biomarkers, we employed both *t*-tests and non-parametric tests (independent *t*-tests: fat mass; HbA1c, HDL, LDL, muscle mass, SBP, TRG; VISA-A, body mass; Mann–Whitney U tests: cholesterol, CRP, DBP, IL-6, TNF-α, VAS). Continuous variables were described using means and standard deviations (SD), while categorical variables were presented as percentages.

We applied both Intention-to-Treat (ITT) and Per-Protocol (PP) analyses to evaluate the study outcomes. The ITT analysis included all participants who were randomized and assigned to a treatment group, regardless of adherence or follow-up status; it served as the primary analysis to preserve randomization and minimize bias. In contrast, the PP analysis focused on participants who completed the 12-week intervention and had both pre- and post-intervention data available; it was used as a sensitivity analysis.

For normally distributed outcomes, between-group effect sizes are reported as Cohen’s d with 95% confidence intervals (95% CI), based on independent-samples *t*-tests. For outcomes not meeting normality assumptions, effect sizes are reported as Rosenthal’s r with 95% CIs, based on Mann–Whitney U tests [35,36].

To address missing data, we used multiple imputation by chained equations, assuming data were missing at random [37]. Sensitivity analyses were performed to examine the impact of data imputation by comparing results from datasets with and without imputed values, thus exploring the robustness of our findings.

Secondary outcomes were analyzed in the same between-group framework but were considered exploratory, given the pilot trial’s sample size. Within-group pre–post changes are reported only as descriptive supplements and do not constitute primary efficacy evidence.

## 3. Results

Overall, 26 individuals expressed interest in the study, of whom 16 met the inclusion criteria and were enrolled in the analysis (Figure 1). There were no significant differences in the anthropometric characteristics of the two groups at baseline (Table 1 and Table 2). Among all participants, nine reported left Achilles tendon pain, three reported right-sided pain, and four experienced bilateral pain.

### 3.1. Primary Outcomes

At baseline, there were no significant differences between the groups in VISA-A (*p* = 0.093) or VAS (*p* = 0.632) scores. The primary between-group analysis revealed no significant post-intervention difference in VISA-A scores between the two groups (Table 3).

**Figure 2:**
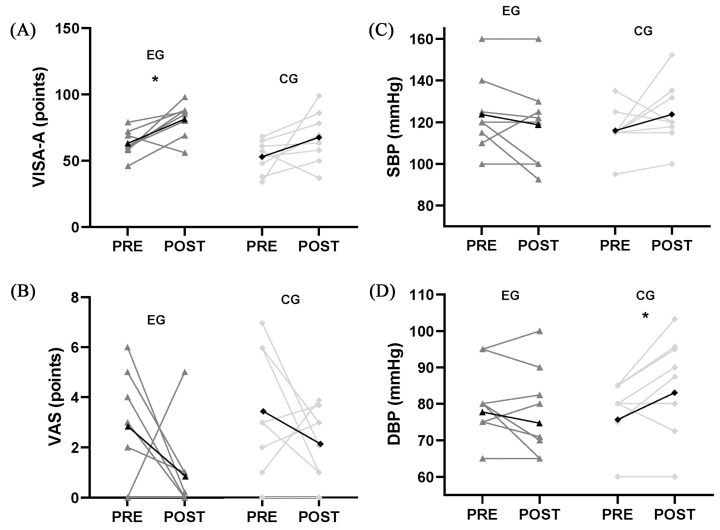
VISA-A scores (**A**), VAS scores (**B**), SBP (**C**), and DBP (**D**) at pre- and post-measurement in the experimental and the control group; * indicates a significant difference between pre- and post-measurement (*p* < 0.05). The black dots represent the mean values, while light gray diamonds represent the individual values in the control group (CG) and dark gray triangles represent the individual values in the experimental group (EG). Abbreviations: DBP: diastolic blood pressure; SBP: systolic blood pressure; VAS: visual analog scale; VISA-A: Victorian Institute of Sport Assessment—Achilles.

From pre- to post-intervention (Figure 2), all participants presented a significant improvement in VISA-A scores after the 12-week intervention (*p* = 0.005, effect size = 0.834, 95% CI = 0.252–1.396), with an average increase of 16.45 ± 15.74 points. The intervention group demonstrated a significant increase in VISA-A scores after 12 weeks, with an average improvement of 18.26 ± 11.90 points (*p* = 0.013, effect size = 1.161, 95% CI = 0.224–2.052), while in the control group, the VISA-A scores did not significantly differ between pre- and post-intervention. VAS scores significantly dropped after the intervention across all participants (*p* = 0.048, effect size = 0.608, 95% CI = 0.220–0.866), with an average reduction of 1.64 ± 2.07 points, although the change was not statistically significant in the independent group analysis. There was no significant difference in VISA-A scores between the two groups after the intervention (Table 3).

Sensitivity analyses (Supplemental File, Tables S3 and S4) revealed that all participants experienced significant improvement in VISA-A scores after the 12-week intervention (*p* = 0.024), with an average increase of 14.82 ± 17.72 points. Group-specific analyses revealed significant improvement only in the intervention group, with an average increase of 13.84 ± 22.32 points (*p* = 0.033). No significant differences in VAS scores were observed before and after the intervention.

### 3.2. Secondary Outcomes

At baseline (Table 2), there were no significant differences between the groups in CRP (*p* = 0.511), IL-6 (*p* = 0.268), TNF-α (*p* = 0.317), TRG (*p* = 0.172), HbA1c (*p* = 0.684), HDL (*p* = 0.457), LDL (*p* = 0.305), total cholesterol (*p* = 0.680), SBP (*p* = 0.347), or DBP (*p* = 0.693). From pre- to post-intervention (Table 2), IL-6 levels significantly improved across all participants (*p* = 0.034). The control group experienced a significant increase of 6.74 ± 12.26 mmHg in DBP (*p* = 0.046, Figure 2). No other biomarkers showed significant changes.

From pre- to post-intervention (Table 2), a nominally significant improvement in IL-6 was observed across all participants (*p* = 0.034, effect size = 0.879, 95% CI = 0.879–0.879). However, no significant between-group difference in IL-6 change was detected, and this isolated finding should be interpreted with caution given the small sample size and the exploratory nature of secondary analyses. The control group experienced a significant increase of 6.74 ± 12.26 mmHg in DBP (*p* = 0.046, effect size = 0.842, 95% CI = 0.594–0.891; Figure 2); this finding was not accompanied by a significant between-group difference. No other biomarkers showed significant changes.

None of the participants reported pre-existing hyperlipidemia, diabetes mellitus, or hypertension. At baseline, one subject (EG) exhibited elevated IL-6 levels (>6 ng/L), while IL-6 levels were in the normal range post-intervention. One participant (EG) demonstrated hypertriglyceridemia (>150 mg/dL) at baseline, while triglyceride levels were in the normal range post-intervention. One participant (EG) showed elevated LDL cholesterol (>116 mg/dL, experimental group) at baseline, while LDL data were missing post-intervention, and two participants (CG&EG) presented with elevated total cholesterol (>190 mg/dL) and both were back to normal range post-intervention. Post-intervention, one individual (EG) presented with increased TNF-α (>15 pg/mL), two (EG &CG) with elevated LDL cholesterol (>116 mg/dL), and one (CG) with high total cholesterol (>190 mg/dL). All other parameters across participants remained within clinically established reference ranges [38].

Sensitivity analyses (Supplemental File, Tables S3 and S4) revealed that there were no significant differences before and after the 12-week intervention for blood biomarkers or blood pressure measurements. No significant differences in blood biomarkers or blood pressure scores were observed between groups after the intervention.

## 4. Discussion

This randomized controlled pilot trial compared nutritional advice combined with home-based exercise training to exercise alone. In contrast to our hypothesis, we were not able to confirm that combining exercise therapy with nutritional advice is more effective than exercise therapy alone in treating Achilles tendinopathy, as no statistically significant differences emerged between groups in VISA-A, VAS, or blood-derived biomarkers. Although the within-group analysis demonstrated a significant VISA-A improvement in the experimental group only, this finding offers no evidence of superiority over exercise alone and must be interpreted with caution given the lack of between-group significance and the small sample size.

The applied exercise intervention demonstrated clinical efficacy, with the exercise protocol’s effectiveness validated in prior studies. A 12-week home-based Achilles tendon training program notably enhanced plantar flexor tendon unit strength and jump performance in healthy individuals [29], as well as Achilles tendon stiffness, tendon cross-sectional area, and VISA-A scores in individuals with Achilles tendinopathy [20]. Furthermore, isometric training has been shown to mitigate central pain facilitation mechanisms [39] and induce both localized and systemic hypoalgesia [40]. However, combining nutritional advice with exercise did not yield superior outcomes compared to exercise alone in our study. Although there is no other evidence of the application of nutritional advice on tendinopathy, findings have emerged in an RCT investigating the effect of exercise therapy combined with nutritional supplements on Achilles tendinopathy. The 3-month intervention program that combined collagen peptide supplementation or placebo with exercise enhanced the patients’ VISA-A scores more significantly in the experimental group, indicating that oral supplementation of specific collagen peptides may accelerate the clinical benefits of exercise therapy [23]. While our study supports the effectiveness of exercise intervention programs, the superiority of a combined program of nutritional advice and exercise intervention still needs further exploration.

In contrast to controlled changes in diet or intake of supplements, standalone dietary counseling may demonstrate limited clinical impact on functional or prognostic outcomes. An intention-to-treat randomized controlled trial involving 76 chronic heart failure patients found that structured nutritional counseling combined with supplementation significantly improved nutritional status versus standard care alone, while no differences were observed in one-year mortality or hospitalization rates [41]. Similar results were observed in a frail population, in which 96 community-dwelling frail older adults aged 75 years and older were given nutritional advice and/or exercise. No differences in resting metabolic rate were observed between intervention groups at 3 months post-intervention and at a 9-month follow-up, and no correlation was observed over time between energy intake, resting metabolic rate, and lean body mass [42]. Small differences and a lack of significant effects of nutritional interventions may be due to the heterogeneity of study populations and a lack of targeted treatments [42]. A controlled change in diet may represent a more impactful strategy than generalized nutritional advice. In obese osteoarthritis patients awaiting surgery, 3-month nutritional meal replacement protocols achieved superior improvements in body mass, body mass index, fat mass, and insulin levels compared to nutritional advice [43]. Similarly, a randomized controlled trial involving overweight or obese participants at high risk of metabolic syndrome found that consuming a high-protein, low-sugar meal replacement for six months was more effective than standard nutritional advice in improving health-related quality of life, with a particularly greater improvement observed in physical health [44]. While generalized nutritional advice may show little effect, targeted nutritional advice has been shown to effectively change biomarkers of health. In patients with type 2 diabetes and abdominal obesity, those who received personalized nutritional guidance at least once a week for 16 weeks showed significant improvements in the International Diet Quality Index, body mass, waist circumference, and HbA1c levels compared to those who received general nutritional advice [45]. While exercise training or controlled changes in diet are more powerful in affecting musculoskeletal health compared to counseling, nutritional advice has the potential to induce small improvements in health.

Our analysis revealed an unanticipated finding: a significant increase in DBP following the exercise therapy in the CG. This observation does not align with the literature, as physical activity is well-established as a beneficial strategy for blood pressure control [46]. For instance, a four-week home-based wall squat intervention among normotensive healthy men resulted in a significant reduction in diastolic blood pressure, primarily mediated by a decrease in resting heart rate [47]. In the present study, the observed change in DBP was not robust to sensitivity analyses: In the PP analysis, the DBP increase was no longer significant (*p* = 0.182); the result was no longer significant when the participant with the highest DBP was excluded (*p* = 0.343). In contrast, DBP in the experimental group remained stable (*p* = 0.363) and non-significant in sensitivity analyses (Supplemental File, Tables S5–S8). Therefore, the increase in DBP associated with exercise intervention requires further validation through long-term follow-up.

Personalized therapeutic approaches, both in terms of nutritional guidance and in terms of exercise treatment programs, may be needed to improve the therapeutic efficacy of tendinopathy treatment. Individual variations in response to exercise interventions may compromise rehabilitation effects. Distinct patterns of “high responders,” “low responders,” and “non-responders” to exercise protocols have been observed previously [48]. Notably, elevated IL-6 levels have been associated with diminished therapeutic efficacy in Achilles tendinopathy management [14]. These findings underscore the need for large-scale, comparative studies employing diverse intervention strategies to identify individual variations and develop more targeted therapeutic approaches for tendinopathy management.

## 5. Limitations

Our pilot trial’s small sample size limits definitive conclusions. It also precluded comparative analyses of treatment efficacy between patients with metabolic disorders and/or inflammatory conditions and those without such comorbidities. Additionally, while we implemented multiple imputation for missing data and sensitivity analyses, the elevated attrition rate introduces potential bias in outcome interpretation. All exploratory analyses should be interpreted accordingly. Given the number of secondary outcomes tested in this small pilot trial, no multiplicity correction was applied, and the isolated nominally significant findings should be interpreted with caution, as they may represent false-positive results. Adherence data were limited to self-reported training diaries, and compliance with nutritional advice was only reinforced by a single follow-up telephone call at Week 6. In addition, our sample may have been healthier than the general population, as we recruited in the capital’s city center and in the sports medicine department, which may have reduced the effect of the nutritional advice. The combination of nutritional advice with exercise therapy may be a more promising, low-cost approach in specific populations suffering from more comorbidities or unhealthier nutritional habits.

## 6. Conclusions

This pilot study explored the therapeutic potential of nutritional advice combined with exercise treatment. The observed improvement in the VISA-A score suggests that the exercise program was associated with improvement over time, whereas this pilot study did not provide clear evidence that adding nutritional advice was superior to exercise alone. Definitive conclusions regarding its superiority in treating Achilles tendinopathy over isolated exercise regimens require validation through larger sample trials with standardized adherence monitoring.

## Figures and Tables

**Table 1:** Baseline characteristics of the participants.

Variable	Experimental	Control	*p*-Value #	Total
Age [year]	38.38 ± 10.03	40.38 ± 9.44	0.688	39.38 ± 9.46
Sex [F/M]	4/4	3/5	0.317	7/9
IPAQ [Met-min/week]	4553 ± 2513	2961 ± 2221	0.201	3757 ± 2434
Height [cm]	172.8 ± 7.5	174.9 ± 15.7	0.739	173.8 ± 11.9
Body mass	66.23 ± 10.87	71.44 ± 12.75	0.394	68.83 ± 11.76
Fat free mass	29.21 ± 6.29	29.21 ± 6.29	0.347	29.21 ± 6.29
Fat mass	13.78 ± 9.60	13.78 ± 9.60	0.721	13.78 ± 9.60

#: For between-group comparisons of baseline characteristics. Independent *t*-tests were performed for continuous variables with a normal distribution (age, height, IPAQ scores, fat-free mass, body mass). Mann–Whitney U tests were used for non-normally distributed continuous variables (fat mass).

**Table 2:** Primary and secondary outcomes pre- and post-intervention.

Variable	Experimental		Effect Size (95% CI)/% Difference	Control		Effect Size (95% CI)/% Difference	Total		Effect Size (95% CI)/% Difference
	Pre	Post		Pre	Post		Pre	Post	
IL-6 [ng/L]	87.5% < 7.0	all < 7.0	12.5%	all < 7.0	all < 7.0	-	93.8% < 7.0	all < 7.0 *	6.2%
TNF-α [pg/mL]	all < 15.0	87.5% < 15.0	12.5%	all < 15.0	75% < 15.0	25%	all < 15.0	81.3% < 15.0	18.7%
CRP [mg/L]	87.5% < 5.0	75% < 5.0	12.5%	all < 5.0	all < 5.0	-	93.8% < 5.0	68.8% < 5.0	25%
HbA1c [%]	5.24 ± 0.35	5.33 ± 0.31	0.326 (−0.398, 1.028)	5.31 ± 0.37	5.49 ± 0.26	0.687 (−0.11, 1.446)	5.28 ± 0.35	5.41 ± 0.29	0.505 (−0.024, 1.020)
HDL [mg/dL]	68.75 ± 12.19	69.95 ± 13.42	0.134 (−0.567, 0.826)	64.75 ± 8.40	70.51 ± 11.85	0.496 (−0.257, 1.219)	66.75 ± 10.32	70.23 ± 12.23	0.339 (−0.171, 0.838)
TRG [mg/dL]	72.25 ± 40.47	79.52 ± 26.78	0.297 (0, 0.891)	77.88 ± 22.31	70.76 ± 9.22	−0.363 (−1.068, 0.367)	75.06 ± 31.70	75.14 ± 19.87	0.003 (−0.487, 0.493)
LDL [mg/dL]	87.75 ± 24.51	101.56 ± 35.54	0.326 (−0.398, 1.028)	99.38 ± 18.83	96.96 ± 7.59	−0.108 (−0.800, 0.591)	93.56 ± 21.95	99.26 ± 24.94	0.169 (−0.328, 0.659)
Cholesterol [mg/dL]	164.50 ± 32.21	166.75 ± 20.53	0.063 (−0.633, 0.754)	169.88 ± 16.29	182.54 ± 32.36	0.347 (0, 0.842)	167.19 ± 24.81	174.64 ± 27.42	0.116 (0.013, 0.582)
Body mass [kg]	66.23 ± 10.87	64.56 ± 9.79	−0.441 (−1.156, 0.302)	71.44 ± 12.75	70.47 ± 13.09	−0.407 (−1.117, 0.330)	68.83 ± 11.76	67.51 ± 11.58	−0.429 (−0.935, 0.091)
Fat free mass [kg]	29.21 ± 6.29	29.13 ± 7.29	−0.015 (−0.707, 0.679)	32.90 ± 8.69	34.56 ± 8.68	0.470 (−0.279, 1.189)	31.06 ± 7.57	31.84 ± 8.24	0.168 (−0.329, 0.659)
Fat mass [kg]	13.78 ± 9.60	12.15 ± 4.73	0.099 (0, 0.842)	12.93 ± 3.82	9.31 ± 3.66	−0.671 (−1.426, 0.122)	13.35 ± 7.07	10.73 ± 4.34	0.323 (0.013, 0.698)

*: *p* < 0.05. Comparisons between pre- and post-intervention were analyzed. For biomarkers provided by the laboratory as categorical ranges, data are presented as the percentage of difference. Abbreviations: CRP: C-reactive protein; IL-6: interleukin-6; HbA1c: hemoglobin/hemoglobin A1c; HDL: high-density lipoprotein; LDL: low-density lipoprotein; TNF-α: tumor necrosis factor alpha; TRG: triglyceride. Reference range of blood indicators: CRP: reference < 5.0; IL-6: reference < 7.0; TNF-α: reference < 15.0.

**Table 3:** Between-group differences in outcomes at post-intervention.

Variables	*p*-Value	Effect Size	95% CI	
			LCI	UCI
Primary outcomes				
VISA-A	0.126	0.814	−0.224	1.825
VAS	0.068	−0.446	−0.814	0.000
Blood outcomes				
IL-6	0.589	−0.092	−0.407	0.210
TNF-α	0.332	−0.184	−0.512	0.118
CRP	0.424	−0.171	−0.551	0.210
HbA1c	0.291	−0.548	−1.539	0.462
HDL	0.931	−0.044	−1.024	0.937
TRG	0.396	0.437	−0.563	1.423
LDL	0.726	0.179	−0.807	1.158
Cholesterol	0.345	−0.583	−1.576	0.430
Blood pressure				
SBP	0.582	−0.282	−1.262	0.709
DBP	0.247	−0.568	−1.560	0.444
Anthropometric information				
BMI	0.227	−0.131	−0.630	0.368
Body mass	0.324	−0.511	−1.500	0.495
Fat-free mass	0.196	−0.678	−1.678	0.344
Fat mass	0.200	0.105	−0.394	0.552

Between-group comparisons were performed, and significance was set when *p*-value < 0.05: Independent *t*-tests: Fat weight; HbA1c, HDL, LDL, muscle weight, SBP, TRG; VISA-A, weight; Mann–Whitney U tests: Cholesterol, CRP, DBP, IL-6, TNF-α, VAS.

## Data Availability

The data that support the findings of this study are available from the corresponding author upon reasonable request due to ethical reasons.

## Supplementary Materials

The following supporting information can be downloaded at https://www.mdpi.com/article/10.3390/nu18101519/s1, Table S1: *p*-Values for *t*-tests or non-parametric tests used to detect differences between pre and post intervention in all participants, the experimental and the control group; Table S2: *p*-Values for *t*-tests or non-parametric tests used to detect differences between baseline variables the experimental and the control group (ITT); Table S3: Primary and secondary outcomes pre and post the intervention (PP); Table S4: *p*-Values for *t*-tests or non-parametric tests used to detect differences between variables in the experimental and the control group post-intervention (PP); Table S5: Results of the sensitivity analysis of DBP in the control group; Table S6: *p*-Values for non-parametric tests used to detect differences of DBP in control group between pre and post intervention (after excluding the individual whose DBP decreased); Table S7: *p*-Values for non-parametric tests used to detect differences of DBP in control group between pre and post intervention (after excluding the individual whose DBP was highest); Table S8: The sensitivity analysis of DBP in the experiment group. CONSORT 2025 checklist of information to include when reporting a randomised trial *. Training Diary.

## Abbreviations

The following abbreviations are used in this manuscript:

CG	control group
CONSORT	Consolidated Standards of Reporting Trials
CRP	C-reactive protein
EG	experimental group
HbA1c	glycated hemoglobin/hemoglobin A1c
HDL	high-density lipoprotein
HSR	heavy slow resistance training
IL-6	interleukin-6
IPAQ	the International physical activity questionnaire
ITT	intention-to-treat
LDL	low-density lipoprotein
PP	per-protocol
SD	standard deviations
TNF-α	tumor necrosis factor alpha
TRG	triglyceride
VAS	visual analog scale
VISA-A	Victorian Institute of Sport Assessment—Achilles
