# Supplementary material for: Can the Effects of Exercise Therapy on Achilles Tendinopathy Be Enhanced by Adding Nutritional Advice—A Randomized Controlled Pilot Study
Source: Nutrients. 2026 May 10;18(10):1519. doi: 10.3390/nu18101519 (PMC13209224; doi:10.3390/nu18101519)
Supplement: Supplementary file 1 [file nutrients-18-01519-s001.zip › nutrients-4285801-supplementary.pdf]

Table S1: P-Values for T-tests or non-parametric tests used to detect differences between pre and post intervention in all participants, the experimental and the control group

**All participants**			
non-parametric tests	**P-value**	***t-tests***	**P-value**
**IL6**	**0.034**	HbA1	0.061
TNFα	0.18	HDL	0.196
CRP	0.157	TRG	0.992
Cholesterol	0.642	LDL	0.51
**VAS**	**0.048**	**VISAA**	**0.005**
Fat mass	0.215	SBP	0.73
		DBP	0.386
		Body mass	0.107
		Fat free mass	0.513
**Group participants**			
**Experiment**			
non-parametric tests	**P-value**	***t-tests***	**P-value**
IL6	0.059	HbA1	0.387
TNFα	1	HDL	0.716
CRP	0.102	LDL	0.388
TRG	0.401	Cholesterol	0.864
Fat mass	0.779	**VISAA**	**0.013**
VAS	0.175	SBP	0.279
		DBP	0.323
		Body mass	0.253
		Fat free mass	0.967
**Control**			
non-parametric tests	**P-value**	***t-tests***	**P-value**
IL6	0.317	HbA1	0.093
TNFα	0.083	HDL	0.203
CRP	1	TRG	0.339
Cholesterol	0.327	LDL	0.768
**DBP**	**0.046**	VAS	0.247
		VISAA	0.129
		SBP	0.223
		Body mass	0.288
		Fat free mass	0.226
		Fat mass	0.1

Abbreviation: CRP: C-reactive protein; DBP: diastolic blood pressure; HbA1c: Hemoglobin/hemoglobin A1c; HDL: high-density lipoprotein; IL-6: Interleukin-6; ITT, intention to treat; LDL: low-density lipoprotein; SBP: systolic blood pressure; TNF-α: Tumor Necrosis Factor alpha; TRG: triglyceride; VAS: visual analog scale; VISA-A: Victorian Institute of Sport Assessment-Achilles.

Table S2.

P-Values for T-tests or non-parametric tests used to detect differences between baseline variables the experimental and the control group (ITT)

Mann-Whitney test	
	**Significance (two-tailed)**
**IL6pre**	0.268
**TNF-αpre**	0.317
**CRPpre**	0.511
**TRGpre**	0.172
**VASpre**	0.632
**Fat mass Pre**	0.674
T test	
	**Significance (two-tailed)**
**HbA1cpre**	0.684
**HDLpre**	0.457
**LDLpre**	0.305
**Cholesterolpre**	0.680
**VISAApre**	0.093
**SBPPre**	0.347
**DBPpre**	0.693
**Body massPre**	0.394
**Fat free weightPre**	0.347

This table presents the difference of variables at baseline between the two groups of subjects after multiple interpolation.

Table S3. Primary and secondary outcomes pre and post the intervention (PP)

Variables	Experimental		Control		Total	
	Pre	Post	Pre	Post	Pre	Post
**IL6**	83.3% < 7.0	< 7.0	< 7.0	< 7.0	90.9% < 7.0	< 7.0
**TNF α**	< 15.0	83.3% < 15.0	< 15.0	< 15.0	< 15.0	90.9% < 15.0
**CRP**	< 5.0	< 5.0	< 5.0	< 5.0	< 5.0	< 5.0
**HbA1c**	5.33±0.35	5.28±0.34	5.44±0.30	5.62±0.24	5.38±0.32	5.44±0.34
**HDL**	69.50±13.00	70.83±9.70	67.40±4.39	71.60±13.01	68.55±9.67	71.18±10.72
**TRG**	79.50±42.88	80.67±31.56	80.20±27.73	68.60±11.50	79.82±35.03	75.18±24.31
**LDL**	91.50±15.50	100.17±41.07	93.80±8.76	98.80±6.61	92.55±12.34	99.55±29.35
**Cholesterol**	170.33±20.15	164.17±23.63	164.40±4.72	187.20±41.95	167.64±14.88	174.64±33.58
**VAS**	2.17±1.94	1.17±1.94	3.40±2.61	1.60±1.34	2.73±2.24	1.36±1.63
**VISAA**	66.83±7.86	80.67±12.45*	50.00±13.98	66.00±25.74	59.18±13.65	74.00±20.03*
**SBP**	125.00±21.68	122.50±22.30	117.00±14.83	113.75±9.46	121.36±18.45	119.00±18.07
**DBP**	81.67±11.69	78.33±14.38	76.00±9.62	81.25±15.48	79.09±10.68	79.50±14.03
**Body mass**	64.10±10.48	64.05±10.83	71.32±13.93	71.36±13.68	67.38±12.12	67.37±12.17
**Fat free mass**	29.62±7.14	29.48±7.11	33.28±9.07	34.04±8.09	31.28±7.88	31.55±7.56
**Fat mass**	10.97±3.57	11.17±3.33	12.28±3.33	11.02±1.51	11.56±3.36	11.10±2.54

*: $P < 0.05$. Comparisons between pre- and post-intervention were analyzed.

This table presents the outcomes of the subjects that conform to the Per-protocol analysis before and after the intervention.

Abbreviation: PP: Per-Protocol.

Reference range of blood indicators: CRP: reference: < 5.0; IL-6: references: < 7.0; TNF-α: reference: < 15.0.

Table S4. P-Values for T-tests or non-parametric tests used to detect differences between variables in the experimental and the control group post-intervention (PP)

Variable (post)	*P*-value
Mann-Whitney U	
**IL6**	0.892
**TNFα**	1
**CRP**	0.399
**LDL**	0.584
T test	
**HbA1c**	0.098
**HDL**	0.913
**TRG**	0.441
**Cholesterol**	0.279
**VAS**	0.684
**VISAA**	0.246
**SBP**	0.486
**DBP**	0.768
**Weight**	0.347
**Muscle weight**	0.346
**Fat weight**	0.930

This table presents the difference of variables at post-intervention between the two groups of subjects, without multiple interpolation.

Table S5. Results of the sensitivity analysis of DBP in the control group

Paired Samples Test								
	Paired Differences					t	df	Sig. (2-tailed)
	Mean	Std. Deviation	Std. Error Mean	Paired Differences — 95% Confidence Interval of the Difference				
				Lower	Upper			
**DBPpre – DBPpost**	-5.00000	5.77350	2.88675	-14.18693	4.18693	-1.732	3	**.182**

Table S6. P-Values for non-parametric tests used to detect differences of DBP in control group between pre and post intervention (after excluding the individual whose DBP decreased)

Wilcoxon Signed Ranks Test	
	DBP post - DBP pre
Z	-2.032
Sig. (2-tailed)	.042

Table S7. P-Values for non-parametric tests used to detect differences of DBP in control group between pre and post intervention (after excluding the individual whose DBP was highest)

Wilcoxon Signed Ranks Test	
	DBP post - DBP pre
Z	-0.948
Sig. (2-tailed)	.343

Table S8. The sensitivity analysis of DBP in the experiment group:

Paired Samples Test

	Paired Differences					t	df	Sig. (2-tailed)
	Mean	Std. Deviation	Std. Error Mean	Paired Differences — 95% Confidence Interval of the Difference				
				Lower	Upper			
**DBPpre – DBPpost**	3.33333	8.16497	3.33333	-5.23527	11.90194	1.000	5	**.363**

CONSORT 2025 checklist of information to include when reporting a randomised trial*

Section / Topic	No	CONSORT 2025 checklist item description	Reported on page no.
**Title and abstract**			**1**
Title and structured abstract	1a	Identification as a randomised trial	1
	1b	Structured summary of the trial design, methods, results, and conclusions	1
**Open science**			
Trial registration	2	Name of trial registry, identifying number (with URL) and date of registration	1
Protocol and statistical analysis plan	3	Where the trial protocol and statistical analysis plan can be accessed	9
Data sharing	4	Where and how the individual de-identified participant data (including data dictionary), statistical code and any other materials can be accessed	9
Funding and conflicts of interest	5a	Sources of funding and other support (e.g., supply of drugs), and role of funders in the design, conduct, analysis and reporting of the trial	9
	5b	Financial and other conflicts of interest of the manuscript authors	9
**Introduction**			

Background and rationale	6	Scientific background and rationale	2
Objectives	7	Specific objectives related to benefits and harms	3
**Methods**			**3**
Patient and public involvement	8	Details of patient or public involvement in the design, conduct and reporting of the trial	3
Trial design	9	Description of trial design including type of trial (e.g., parallel group, crossover), allocation ratio, and framework (e.g., superiority, equivalence, non-inferiority, exploratory)	4
Changes to trial protocol	10	Important changes to the trial after it commenced including any outcomes or analyses that were not prespecified, with reason	4
Trial setting	11	Settings (e.g., community, hospital) and locations (e.g., countries, sites) where the trial was conducted	4
Eligibility criteria	12a	Eligibility criteria for participants	3
	12b	If applicable, eligibility criteria for sites and for individuals delivering the interventions (e.g., surgeons, physiotherapists)	3
Intervention and comparator	13	Intervention and comparator with sufficient details to allow replication. If relevant, where additional materials describing the intervention and comparator (e.g., intervention manual) can be accessed	4
Outcomes	14	Pre-specified primary and secondary outcomes, including the specific measurement variable (e.g., systolic blood pressure), analysis metric (e.g., change from baseline, final value, time to event), method of aggregation (e.g., median, proportion), and time point for each outcome	5
Harms	15	How harms were defined and assessed (e.g., systematically, non-systematically)	4

Sample size	16a	How sample size was determined, including all assumptions supporting the sample size calculation	5
	16b	Explanation of any interim analyses and stopping guidelines	5
Randomisation:			4
Sequence generation	17a	Who generated the random allocation sequence and the method used	4
	17b	Type of randomisation and details of any restriction (e.g., stratification, blocking and block size)	4
Allocation concealment mechanism	18	Mechanism used to implement the random allocation sequence (e.g., central computer/telephone; sequentially numbered, opaque, sealed containers), describing any steps to conceal the sequence until interventions were assigned	4
Implementation	19	Whether the personnel who enrolled and those who assigned participants to the interventions had access to the random allocation sequence	4
Blinding	20a	Who was blinded after assignment to interventions (e.g., participants, care providers, outcome assessors, data analysts)	4
	20b	If blinded, how blinding was achieved and description of the similarity of interventions	4
Statistical methods	21a	Statistical methods used to compare groups for primary and secondary outcomes, including harms	5
	21b	Definition of who is included in each analysis (e.g., all randomised participants), and in which group	5
	21c	How missing data were handled in the analysis	5
	21d	Methods for any additional analyses (e.g., subgroup and sensitivity analyses), distinguishing prespecified from post-hoc	5

**Results**			**5**
Participant flow, including flow diagram	22a	For each group, the numbers of participants who were randomly assigned, received intended intervention, and were analysed for the primary outcome	5
	22b	For each group, losses and exclusions after randomisation, together with reasons	5-6
Recruitment	23a	Dates defining the periods of recruitment and follow-up for outcomes of benefits and harms	6
	23b	If relevant, why the trial ended or was stopped	6
Intervention and comparator delivery	24a	Intervention and comparator as they were actually administered (e.g., where appropriate, who delivered the intervention/comparator, how participants adhered, whether they were delivered as intended [fidelity])	6
	24b	Concomitant care received during the trial for each group	6
Baseline data	25	A table showing baseline demographic and clinical characteristics for each group	5
Numbers analysed, outcomes and estimation	26	For each primary and secondary outcome, by group:<br>● the number of participants included in the analysis<br>● the number of participants with available data at the outcome time point<br>● result for each group, and the estimated effect size and its precision (such as 95% confidence interval)<br>● for binary outcomes, presentation of both absolute and relative effect size	5
Harms	27	All harms or unintended events in each group	4-5
Ancillary analyses	28	Any other analyses performed, including subgroup and sensitivity analyses, distinguishing pre-specified from post-hoc	5

**Discussion**			**7**
Interpretation	29	Interpretation consistent with results, balancing benefits and harms, and considering other relevant evidence	7
Limitations	30	Trial limitations, addressing sources of potential bias, imprecision, generalisability, and, if relevant, multiplicity of analyses	8

Trainingstagebuch (Training Diary)

Setzen Sie ein Kreuz, wenn Sie das Training für diesen Tag absolviert haben. Eine Trainingseinheit umfasst fünf Sätze. Ein Satz beinhaltet vier Wiederholungen (eine Wiederholung= 3sec. Spannung halten). Zwischen jedem Satz erfolgt eine 1-minütige Pause (Beispiel: Montag/ 5x4 Wdh.).
Im Feld *Schmerz* tragen Sie die Schmerzhöhe, zwischen 0 bis 10, nach dem Training ein (Beispiel: 3/10). Zusätzlich tragen Sie im Feld *Sport/Bewegung* ein, wieviel Minuten Sie am betreffenden Tag sportlich aktiv waren. Im Feld *Notizen* tragen Sie besondere Vorkomnisse ein, die Ihr Training möglicherweise beeinflusst haben (Beispiele: Krankheit, Zerrung, Wettkampf, Erschöpfung, Arbeitsstress, etc.)

Woche 1

Wochentag	Sätze	Schmerz	Sport/Bewegung	Notizen
Mo. ❑				
Di. ❑				
Mi. ❑				
Do. ❑				
Fr. ❑				
Sa. ❑				
So. ❑				

Woche 2

Wochentag	Sätze	Schmerz	Sport/Bewegung	Notizen
Mo. ❑				
Di. ❑				
Mi. ❑				
Do. ❑				
Fr. ❑				
Sa. ❑				
So. ❑				

Woche 3

Wochentag	Sätze	Schmerz	Sport/Bewegung	Notizen
Mo. ❑				
Di. ❑				

Mi. ❑				
Do. ❑				
Fr. ❑				
Sa. ❑				
So. ❑				

Woche 4

Wochentag	Sätze	Schmerz	Sport/Bewegung	Notizen
Mo. ❑				
Di. ❑				
Mi. ❑				
Do. ❑				
Fr. ❑				
Sa. ❑				
So. ❑				

Woche 5

Wochentag	Sätze	Schmerz	Sport/Bewegung	Notizen
Mo. ❑				
Di. ❑				
Mi. ❑				
Do. ❑				
Fr. ❑				
Sa. ❑				
So. ❑				

Woche 6

Wochentag	Sätze	Schmerz	Sport/Bewegung	Notizen
Mo. ❑				
Di. ❑				
Mi. ❑				
Do. ❑				

Fr. ❑				
Sa. ❑				
So. ❑				

Woche 7

Wochentag	Sätze	Schmerz	Sport/Bewegung	Notizen
Mo. ❑				
Di. ❑				
Mi. ❑				
Do. ❑				
Fr. ❑				
Sa. ❑				
So. ❑				

Woche 8

Wochentag	Sätze	Schmerz	Sport/Bewegung	Notizen
Mo. ❑				
Di. ❑				
Mi. ❑				
Do. ❑				
Fr. ❑				
Sa. ❑				
So. ❑				

Woche 9

Wochentag	Sätze	Schmerz	Sport/Bewegung	Notizen

Mo. ❑				
Di. ❑				
Mi. ❑				
Do. ❑				
Fr. ❑				
Sa. ❑				
So. ❑				

Woche 10

Wochentag	Sätze	Schmerz	Sport/Bewegung	Notizen
Mo. ❑				
Di. ❑				
Mi. ❑				
Do. ❑				
Fr. ❑				
Sa. ❑				
So. ❑				

Woche 11

Wochentag	Sätze	Schmerz	Sport/Bewegung	Notizen
Mo. ❑				
Di. ❑				
Mi. ❑				
Do. ❑				
Fr. ❑				
Sa. ❑				
So. ❑				

Woche 12

Wochentag	Sätze	Schmerz	Sport/Bewegung	Notizen
Mo. ❑				
Di. ❑				
Mi. ❑				
Do. ❑				
Fr. ❑				
Sa. ❑				
So. ❑				
